# Dermoscopic Patterns of Acquired Reactive Perforating Collagenosis

**DOI:** 10.5826/dpc.1101a85

**Published:** 2020-12-07

**Authors:** Ömer Faruk Elmas, Asuman Kilitci, Belkiz Uyar

**Affiliations:** 1 Department of Dermatology, Faculty of Medicine, Kirşehir Ahi Evran University, Kirşehir, Turkey; 2 Department of Pathology, Faculty of Medicine, Kirşehir Ahi Evran University, Kirşehir, Turkey

**Keywords:** dermoscopy, perforating dermatosis, perforating collagenosis, transepidermal elimination

## Introduction

Acquired perforating dermatosis (APD) is a cutaneous disorder characterized by transepidermal elimination of dermal connective tissue. Acquired reactive perforating collagenosis (ARPC), the most common form of APD, is usually associated with underlying diabetes mellitus and chronic renal failure. Here we aimed to demonstrate dermoscopic patterns of ARPC in a series of 7 patients.

## Case Presentation

**Figure 1:**
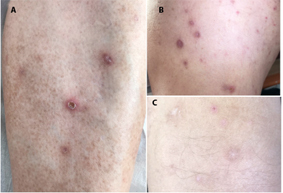
Acquired reactive perforating collagenosis with different stages of lesions. (A) Crusted and (B) excoriated papules and (C) white-to-brown macules.

**Figure 2:**
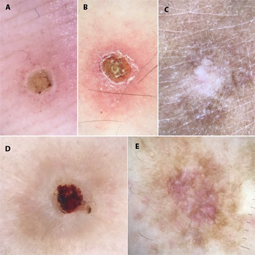
Different dermoscopic patterns of acquired reactive perforating collagenosis: (A) pattern 1; (B) pattern 2; (C) pattern 3; (D) pattern 4; and (E) pattern 5.

**Figure 3:**
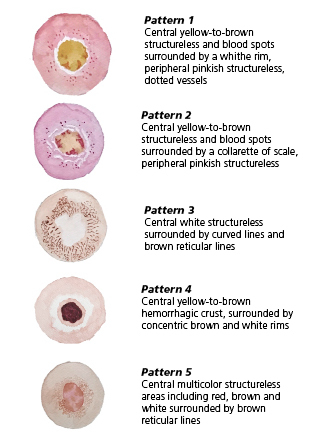
Illustration of the dermoscopic patterns identified for acquired reactive perforating collagenosis.

**Figure 4:**
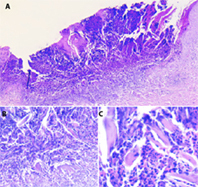
Acquired reactive perforating collagenosis. (A) A cup-shaped epidermal invagination with keratin and inflammatory debris (H&E, ×50) and (B, C) transepidermal elimination of vertically oriented altered collagen (H&E, ×200; ×400).

**Figure 5:**
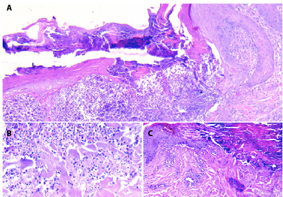
Acquired reactive perforating collagenosis. Hyperkeratosis overlying the cup-shaped depression in the epidermis may correspond to central yellow-to-brown structureless area observed on dermoscopic examination. Epidermal hyperplasia adjacent to the cup-shaped depression with overlying hyperkeratosis may reflect (A) dermoscopic peripheral white rim and collarette scale (H&E, ×50). (B) Transepidermal elimination of altered collagen (H&E, ×400) and (C) superficial dermal fibrotic collagen bundles (H&E, ×200) may correspond to dermoscopic white structureless areas. (C) Superficial dermal vessels and perivascular inflammatory infiltration may represent dermoscopic peripheral pinkish structureless areas and dotted vessels.

A total of 60 lesions from 7 patients with a histopathological diagnosis of ARPC were evaluated. The mean age of the patients was 56 years and the majority was male (n=6). The most common clinical manifestations were itchy, crusted papules (Figure 1A) followed by excoriated papules (Figure 1B) and white-to-brown macules (Figure 1C). The mean disease duration was 10 months. The most common localization of the lesions was on the lower extremities (n=7). Four patients had generalized lesions involving the lower extremities and trunk. All recruited patients had diabetes mellitus. Diabetes mellitus was accompanied by renal failure in one of the patients. A total of 5 different dermoscopic patterns were identified (Figure 2) and illustrated (Figure 3). Pattern 1 (n=14, 23%) showed a white rim surrounding a central yellow-to-brown structureless area. The most common pattern was pattern 2 (n=21, 35%), which was composed of a central yellow–to-brown structureless area and blood spots surrounded by a collarette of scale, a peripheral pinkish structureless area, and dotted vessels. Pattern 3 (central white structureless area surrounded by curved lines and brown reticular lines; n=10, 17%) and pattern 5 (central multicolor structureless areas including red, brown and white areas, surrounded by brown reticular lines; n=7, 12%) were characteristic of the late and healed lesions. Pattern 4 (n=8, 13%) was characterized by central blood spots and concentric white-to-brown rims possibly associated with scratching of the lesions. Four patients showed more than 1 pattern. Histopathological examination of all samples revealed an invaginating epidermal process composed of hyperkeratosis overlying a cup-shaped depression and transepidermal elimination of eosinophilic altered collagen fibers (Figures 4 and 5).

## Conclusions

There are very few reports on the dermoscopic features of ARPC [1,2]. In this case study, we identified a total of 5 different dermoscopic patterns. Pattern 1 and pattern 2 possibly indicate well-established lesions, and the dermoscopic findings represented by these patterns were similar to those reported in previous studies [1,2]. Patterns 4 and 5 have apparently not been identified in previous studies. In this study, only well-established lesions (patterns 1 and 2) were biopsied to achieve a definitive diagnosis, and all specimens showed typical histopathological features of the entity. The histopathological counterpart of the central yellow-to-brown structureless area is central keratin debris. The peripheral white rim and collarette of scale probably reflect invaginating epidermal hyperplasia. The possible histopathological counterpart of the central white structureless area is fibrotic collagen formation in the dermis. The peripheral pinkish structureless area and dotted vessels may represent dermal inflammatory reaction with superficial dilated vessels. Peripheral brown reticular lines may correspond to hyperpigmented basal keratinocytes.

To conclude, dermoscopy may be a useful diagnostic tool in suspected cases of ARPC. The peculiar dermoscopic patterns we identified may lead the way to more comprehensive studies.
